# A Bayesian re-assessment of two Phase II trials of gemcitabine in metastatic nasopharyngeal cancer

**DOI:** 10.1038/sj.bjc.6600199

**Published:** 2002-03-18

**Authors:** S-B Tan, D Machin, B-C Tai, K-F Foo, E-H Tan

**Affiliations:** Division of Clinical Trials and Epidemiological Sciences, National Cancer Centre Singapore, 11 Hospital Drive, Singapore 169610; Clinical Trials Research Unit, Institute of Primary Care and General Practice, University of Sheffield, Sheffield S5 7AU, UK; NMRC Clinical Trials and Epidemiology Research Unit, 10 College Road, Singapore 169851; Department of Medical Oncology, National Cancer Centre Singapore, 11 Hospital Drive, Singapore 169610

**Keywords:** Bayesian, Phase II clinical trial, gemcitabine, nasopharyngeal carcinoma

## Abstract

The Simon two-stage minimax design is a popular statistical design used in Phase II clinical trials. The analysis of the data arising from the design typically involves the use of frequentist statistics. This paper presents an alternative, Bayesian, approach to the design and analysis of Phase II clinical trials. In particular, we consider how a Bayesian approach could have affected the design, analysis and interpretation of two parallel Phase II trials of the National Cancer Centre Singapore, on the activity of gemcitabine in chemotherapy-naïve and in previously treated patients with metastatic nasopharyngeal carcinoma. We begin by explaining the Bayesian methodology and contrasting it with the frequentist approach. We then carry out a Bayesian analysis of the trial results. The conclusions drawn using the Bayesian approach were in general agreement with those obtained from the frequentist analysis. However they had the advantage of allowing for different and potentially more useful interpretations to be made regarding the trial results, as well as for the incorporation of external sources of information. In particular, using a Bayesian trial design, we were able to take into account the results of the parallel trial results when deciding whether to continue each trial beyond the interim stage.

*British Journal of Cancer* (2002) **86**, 843–850. DOI: 10.1038/sj/bjc/6600199 www.bjcancer.com

© 2002 Cancer Research UK

## 

There are many stages in the process of developing an anti-tumour drug and one key stage is the assessment of efficacy in Phase II trials. This is usually demonstrated by using tumour response as the indicator. To this end many thousands of Phase II trials have been conducted. In particular during the ongoing development and assessment of gemcitabine (a fluoride–substituted anti-metabolite) many such trials have already been published (see for example Lund *et al*, 1994), including those that will be the main focus of this paper (Foo *et al*, 2002), and many more are in progress. In general each trial is addressing essentially the same type of question (tumour response) but in a wide variety of disease, patient types and in combination therapies.

The basic designs (in the statistical sense) vary from the informal with no justification of trial size given (see for example, Giralt *et al*, 1997), to the popular two stage Gehan (1961) design (see for example, Scheithauer *et al*, 1999) and the more recent designs as, for example, proposed by Simon (1989). The corresponding analysis of the data arising from these designs is detailed below but is essentially straightforward.

In contrast to the above so-called frequentist designs, several Bayesian designs have been proposed for Phase II trials (see for example, Thall and Simon, 1994; Heitjan, 1997; Stallard, 1998). In this paper, we give a brief overview of the Bayesian approach and show how it could have affected the interpretation of a report of two parallel Phase II trials, conducted at the National Cancer Centre Singapore, on the activity of gemcitabine in patients with metastatic nasopharyngeal carcinoma (NPC).

## MATERIALS AND METHODS

### Phase II trials of gemcitabine

The trials investigated the activity of gemcitabine in chemotherapy-naïve as well as in previously treated patients with metastatic NPC and were run in parallel, with a common protocol. Patients with ECOG performance status ⩽2, adequate renal, hepatic, and bone marrow function, and radiologically measurable NPC, were eligible. All patients were given a maximum of six cycles of gemcitabine at a dose of 1250 mg m^−2^ on days 1 and 8 of a 21-day cycle.

The primary objective in each of these trials was to evaluate the tumour response. ‘Response’ was defined as the sum of both partial and complete responses. Patients who were not evaluable were regarded as failures (non-responders) for the purpose of efficacy determination. Patients who were chemotherapy-naïve and those who had prior chemotherapy were registered, by means of a telephone call through the Clinical Trials and Epidemiology Research Unit, National Medical Research Council, Singapore.

Full details of the background and results from the two trials are given in Foo *et al* (2002).

### Statistical methods

#### Frequentist design

The sample size was estimated separately for each trial assuming a one-sided test size of 5% and a power of 80%. As part of the Simon (1989) two-stage minimax design, it was necessary to specify, for each of the patient groups, a ‘desired overall response rate’ (*R*_1_) as well as a ‘no further interest response rate’ (*R*_0_).

Chemotherapy-naïve patients were enrolled in the first trial (Trial C), which assumed a desired overall response rate of 30% (*R*_1_=0.30), and a no further interest response rate of 10% (*R*_0_=0.10). Using the Simon two-stage minimax design, 15 patients were to be recruited into Stage 1 requiring at least two responses before proceeding to the recruitment of an additional 10 patients in Stage 2. Efficacy was to be claimed if six or more responses were observed from the total of 25 patients.

Similarly patients who had been previously treated with chemotherapy were enrolled in the second trial (Trial P), which assumed an overall response of interest to be 20% (*R*_1_=0.20), and no further interest in gemcitabine if the response was as low as 5% (*R*_0_=0.05). The Simon minimax two-stage design indicated the recruitment of 13 patients in Stage 1 requiring at least one response before proceeding to Stage 2 with a further recruitment of 14 patients. Efficacy was to be claimed if four or more responses were observed from the total of 27 patients.

#### Frequentist analysis

The (frequentist) analysis of response rate from the Phase II trial is straightforward in that the number of responses observed, *r*, is divided by the number of patients recruited, *N*. Thus the observed response rate is 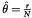 which is an estimate of the true population response rate θ. The distribution of θ is Binomial, which has a likelihood proportional to


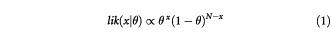


where the *x*'s are the possible number of responses in the Phase II trial which can take any integer value from 0 to *N*.

Although by no means always calculated, the corresponding 95% confidence interval (CI) for θ is often estimated by 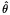 −1.96×s.e.(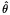) to 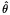
+1.96×s.e.(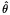). However, this expression is applicable only for relatively large *N* which is not usually the case for Phase II trials. In these circumstances, Newcombe and Altman (2000) describe better methods. They also point out that even if no responses are observed, that is *r*=0, there is still indeed a 95% CI for θ which is from 0 to 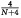.

#### Bayesian design and analysis

The foundation of this approach is Bayes' theorem, which can be expressed as


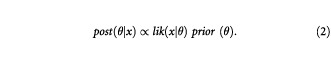


Here the *lik*(*x*|θ) of equation (1) is multiplied by the prior distribution, *prior* (θ), to obtain the posterior distribution, *post* (θ|*x*). The *prior* (θ) summarises what we know about θ before the trial commences and as previously, *lik*(*x*|θ) describes the data to be collected from the trial itself. Finally *post* (θ|*x*) summarises all we know about θ once the trial is completed.

The prior distribution is assumed to be of the form


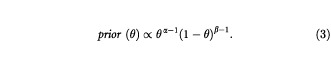


This is a Beta distribution with parameters α and β, which can take any positive real value. When α and β are integers, such a distribution corresponds to a prior belief equivalent to having observed α responses out of a hypothetical *T*=(α+β) patients. This is then similar to the situation modelled by the binomial distribution (see equation (1)), in which we have *x* as the number of responses from *N* patients.

Using equations (1) and (3) in equation (2) results in a posterior distribution of the form


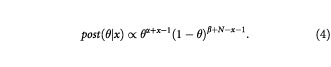


Comparing this with equation (3), we see that this too is a Beta distribution, but of the form Beta (α+*x*, β+*N−x*).

As mentioned previously, the posterior distribution represents our overall belief at the close of the trial about the distribution of the population parameter, θ. Once we have obtained the posterior distribution, we can calculate the exact probabilities of θ being in any region of interest or obtain summary statistics such as its mean value.

#### Priors

The prior distribution summarises the information on θ before the trial commences. We make use of four such types of distributions – clinical, reference, sceptical and enthusiastic. The general way in which each of these are derived is as follows.

The shape of a Beta distribution is dependent on the values of the parameters α and β, and each of the priors will have particular values associated with them. However, eliciting values for α and β is typically not an easy process. Instead, it is often much easier to obtain values for the mean (*M*) and variance (*V*) of the corresponding prior distribution. Once obtained, these values can then be used to obtain α and β by solving the simultaneous equations


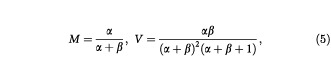


which give


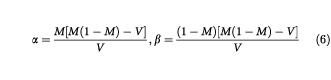


#### Clinical prior

This represents the combined prior belief of informed experts. This will include the subjective prior opinions of the trial investigators and/or other experts, as well as the results of previous similar studies.

Before the start of the trials, no plans were made to carry out a Bayesian analysis of the results. As such, no prior opinion was elicited from the investigators. There were also no published reports on the effect of gemcitabine on tumour response in metastatic NPC patients from which we could derive a clinical prior. However, the investigators were asked for their opinions on the values of *R*_0_ and *R*_1_. For our clinical prior, we then assumed that the investigators' prior belief was such that there was equal probability (here ⅓) of θ being below *R*_0_, between *R*_0_ and *R*_1_, and above *R*_1_. Using the *pbeta* and *qbeta* functions in the S-Plus statistics software (S-Plus 2000 Professional Release 1, MathSoft Inc), appropriate values for α and β were then calculated to define such a Beta distribution.

In mathematical terms, this was done by finding the α and β values that satisfy the integral equation:


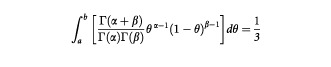


for (*a*, *b*) taking the successive values (0, *R*_0_), (*R*_0_, *R*_1_) and (*R*_1_, 1).

#### Reference prior

A reference or non-informative prior corresponds to the situation when no or very little prior information is available. In practice, this will not often be the case, since the trial investigators are likely to have at least some opinion on the effect of the treatment. Nevertheless, reference priors are useful particularly when we want to focus on just the trial data alone. In this respect, using a reference prior in a Bayesian analysis is similar to carrying out a frequentist analysis, although the manner in which the data is analysed and reported is still very different.

We chose our reference prior distribution to be a uniform (that is ‘flat’) distribution over the entire real line. This corresponds to a state of complete uncertainty.

#### Sceptical prior

A sceptical prior represents the beliefs of individuals who are reluctant to accept that high response rates are possible. Such a prior would be useful for guarding against the possibility that the trial investigators were too optimistic in their expectations.

We defined our sceptical prior to have a Beta distribution with a mean fixed to be *R*_0_ while the probability of exceeding *R*_1_ is set to 0.05, where the small probability of 0.05 is arbitrarily chosen.

#### Enthusiastic prior

An enthusiastic prior is the opposite of a sceptical prior and represents the beliefs of individuals who already accept that the new treatment being tested is promising enough to warrant testing in a Phase III trial. Such a prior would be useful, for example, in the situation where we wish to ensure that potentially good drugs are not rejected on the basis of a handful of ‘not good enough’ results.

We chose our enthusiastic prior to have a Beta distribution with a mean fixed to be *R*_1_ while the probability of being below *R*_0_ is set to 0.05.

### Combining the two trials

From a frequentist viewpoint the two trials considered, although conducted in parallel, are regarded as entirely independent of each other. Thus, the decision to proceed from Stage 1 to Stage 2 in each trial was made without reference to the other. However, there is a detailed record of when the information on response became available for each patient, and so we can illustrate (at least in part) how using Bayesian methods, they may have been considered jointly. To do this we assume, that before proceeding into Stage 2 of either trial, the response information is reviewed so that instead of automatically implementing the Simon rule, the current posterior distribution of θ for the respective trial is calculated (taking into account the information available from both trials). If this suggests that θ has a relatively high probability (say, ⩾0.25) of exceeding *R*_1_ then Stage 2 is implemented else the particular trial stops at the completion of Stage 1. We refer to 0.25 as the threshold probability and denote it by λ.

The above assumes that data from both patient groups are exchangeable in the sense that they contribute an equal weight of information to the response. This is not likely to be valid as it implies that only one Phase II trial would be necessary in patients with NPC and no particular note taken of their prior treatment history. Consequently, a weighting is required whereby the information (as summarised by the posterior distribution) from one group is adjusted downwards if it is being used to modify the prior of the other. The assumption here is that (say) a ‘satisfactory’ response rate in one group may indicate that a ‘satisfactory’ response rate is likely to be observed in the other but the rates in each group may not be the same.

This down weighting can be done by increasing the variance of the posterior distribution but retaining the same mean value. This is accomplished by replacing *V* in equation (6) by *kV*, where *k*>1. This reduces the values of both α and β and is analogous to reducing the number of patients on which the mean and variance are based.

## RESULTS

### Trial outcome and frequentist analysis

A total of 52 patients (25 chemotherapy-naïve, 27 previously treated with prior chemotherapy) were enrolled between January and November 1999.

Of the 15 chemotherapy-naïve patients recruited in Stage 1 of Trial C, three achieved a response. The recruitment of a further 10 patients as specified in Simon's design was thus initiated in Stage 2. Of these, four achieved a response. The overall response to gemcitabine among the 25 patients of Trial C was thus 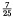 or 28% (95% CI: 14 to 48%). This just exceeded the 24% threshold needed to claim efficacy as specified in the design.

 Of the 13 previously treated patients recruited in Stage 1 of Trial P, seven achieved a response. This led to a subsequent recruitment of 14 more patients in Stage 2, with six additional responses being observed in this group. The overall response to gemcitabine among the 27 patients of Trial P was thus 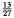 or 48% (95% CI: 31 to 66%), which well exceeded the 15% threshold needed to claim efficacy.

### Bayesian analysis – chemotherapy-naïve group (C)

#### Analysis

**Figure 1:**
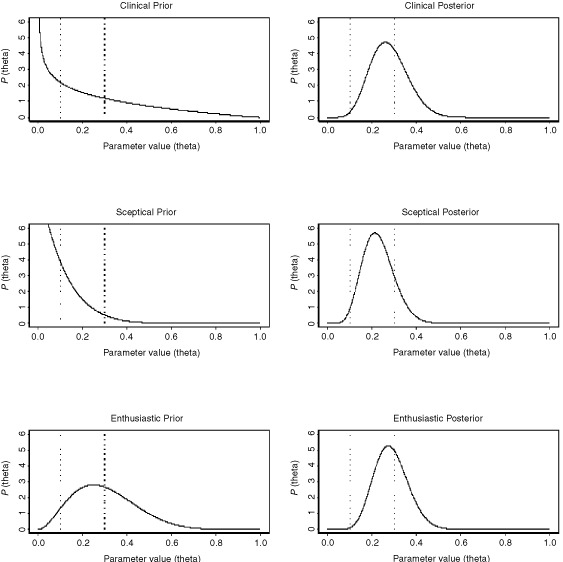
Prior and Stage 2 posterior distributions for the chemotherapy-naïve group.

The prior and Stage 2 posterior distributions are given in Figure 1

. The results are summarised in Table 1

**Table 1:**
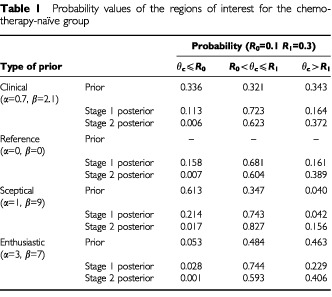
Probability values of the regions of interest for the chemo therapy-naïve group

.

**Figure 2:**
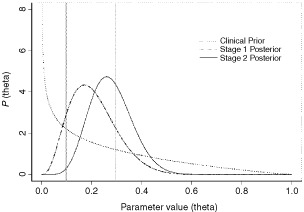
Clinical prior, Stage 1 and Stage 2 posterior for the chemotherapy-naïve group.

Using the clinical prior distribution of Beta (0.7, 2.1), the posterior distribution for the chemotherapy-naïve group after Stage 1 is now Beta (0.7+3, 2.1+12) or Beta (3.7, 14.1). This is shown, together with the clinical prior, in Figure 2

. The data from Stage 1 has updated the prior belief by ‘shifting’ the distribution towards the right, so that most of the distribution now falls within the interval *R*_0_=0.1 to *R*_1_=0.3. The associated probability has increased from 0.321 to 0.723 (Table 1). This represents the posterior belief after having taken into account the results of Stage 1. This updated belief is intuitively reasonable since the 20% response (three responses out of 15) from Stage 1 suggests that θ_c_ is likely to fall between the predefined *R*_0_ and *R*_1_.

The 40% response from Stage 2 (four responses from the subsequent 10 patients) leads to a (further updated) posterior distribution Beta (3.7+4, 14.1+6) or Beta (7.7, 20.1). The posterior distribution after Stage 2 as shown in Figure 2 again falls mainly in the interval *R*_0_ to *R*_1_. The distribution is sharper and narrower than that obtained for Stage 1. This reflects the increased certainty associated with the posterior beliefs after Stage 2, due to a larger total amount of data now being available.

We note that although the 40% response in Stage 2 might have suggested that the main bulk of the updated posterior would fall in the interval corresponding to θ_*c*_>0.3, this is not the case and the bulk remains within 0.1<θ_c_⩽0.3. This is because the sample size in Stage 1 is larger than that in Stage 2. Hence, the results from Stage 1 have a larger influence on the final posterior belief than the results from Stage 2.

Thus, under the clinical prior, the final (after Stage 2) posterior distribution suggests that there is a 62% chance (Table 1) of the true response proportion being between *R*_0_=0.1 and *R*_1_=0.3, while the probability that it is greater than *R*_1_ is about 37%. There is only a 1% chance that it will be less than *R*_0_.

#### Interpretation

We recall that *R*_0_ corresponds to the ‘no further interest response rate’ and *R*_1_ to the ‘desired overall response rate’. In which case, the above can be interpreted: that while there is a very strong case for carrying out further investigations of the treatment, the results are not quite as promising as was desired before the start of the trial.

**Figure 3:**
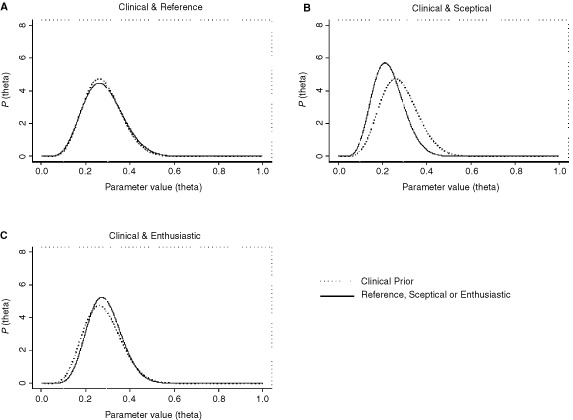
Stage 2 posterior distributions, corresponding to different priors, for the chemotherapy-naïve group.

Using the reference prior, we obtain results that are quite similar to those obtained when we used the clinical prior (see Figure 3A

and Table 1), with for example the Stage 2 probability of θ_c_ exceeding *R*_1_ being 0.389 as opposed to 0.372. The reason for this is that the clinical prior we used is also quite ‘non-informative’, in the sense that it attaches equal probability to θ_c_ being in each of the three regions.

On the other hand, as might be expected, the sceptical prior suggests a distribution for θ_c_ which is ‘to the left’ of that obtained using the clinical prior (Figure 3B). Correspondingly, the Stage 2 value of Prob(θ_c_>*R*_1_) is also less than 0.372 (Table 1, row 9). For the enthusiastic prior, the distribution is ‘to the right’ of that obtained with the clinical prior (Figure 3C) with the Stage 2 value of Prob(θ_c_>*R*_1_) being somewhat greater than 0.372 at 0.406 (Table 1, row 12).

### Bayesian analysis – previously treated group (P)

#### Analysis

**Figure 4:**
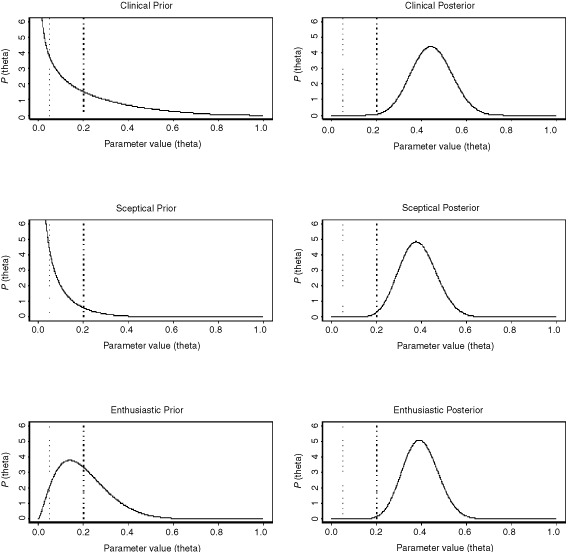
Prior and Stage 2 posterior distributions for the previously treated group.

The prior and Stage 2 posterior distributions are given in Figure 4

. The results are summarised in Table 2

**Table 2:**
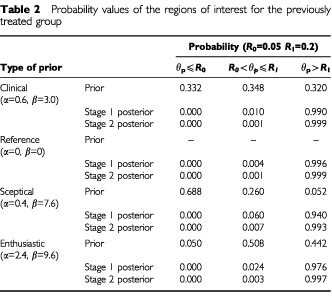
Probability values of the regions of interest for the previously treated group

where all the corresponding Stage 1 and 2 posterior distributions strongly suggest that the true response proportion for these patients (θ_p_) exceeds the *R*_1_=0.2 threshold. This is due to the relatively high number of responses in both Stage 1 (seven out of 13) as well as Stage 2 (six out of 14) observed.

Even with the sceptical prior, the Stage 1 and 2 posteriors still suggest high probabilities, 0.940 and 0.993 respectively, of θ_p_ exceeding *R*_1_.

#### Interpretation

The evidence from the trial data, of θ_p_ being greater than 0.2, is so strong that it is enough to completely change the belief of an individual who, before the start of the trial, felt that there was only a 5% chance of this being possible (as represented by the sceptical prior). In fact, the sceptic's prior belief has been modified by the data to such an extent that he now believes that there is a 99% chance that θ_p_ exceeds 0.2 (Table 2, row 9).

#### Combining the results of both trials

The information with respect to the four stages became available in the order Stage P1, Stage C1, Stage P2 and finally Stage C2. Thus, the first decision to be made is whether to continue to Stage P2 – this will be made without reference to data from Trial C. Using the clinical prior and the data from Stage P1, the resulting posterior distribution with α=7.6, β=9.0 indicates very strongly that Stage P2 should be initiated since the probability that θ_p_ is greater than *R*_1_=0.2 is 0.990 (see Table 2).

The second decision is whether to continue to Stage C2 – however, this will be made in the light of the clinical prior for Trial C together with the Stage C1 and Stage P1 results. Thus the clinical prior for Trial C, will be updated by the data of Stage C1 and then by the (down-weighted) posterior from Stage P1. The first posterior distribution with α=3.7, β=14.1 (from the clinical prior and the data only) indicates only rather weakly that Stage C2 should be initiated since the probability that θ_c_ is greater than the corresponding *R*_1_=0.3 is only 0.164 (see Table 1). However, this is now combined with the down weighted posterior following Stage P1. For illustrative purposes, we choose *k*=4. This results in using a posterior with α=1.6 and β=1.8 which is combined with α=3.7 and β=14.1 to obtain for the second and final posterior distribution α=1.6+3.7=5.3 and β=1.8+14.1=15.9. From this distribution, the probability that θ_c_ is greater than *R*_1_=0.3 is now 0.278. This is (just) above the threshold value of 0.25 (see Discussion) and indicates that Stage C2 should be initiated.

## DISCUSSION

The process of decision making should involve the consideration of evidence from all available sources of information. For example, a physician who wants to make a diagnosis of a patient's condition is unlikely to base the conclusion solely on what is observed during a clinical examination. Instead, account will be taken of the patient's past medical history, along with appropriate blood or other clinical test results. In more complex cases, the physician may also discuss the case with others or consult the medical literature. By analogy, we should not take the results obtained from the clinical trial alone, but place them in the context of other pertinent information.

Bayesian methods attempt to formalise this decision making process. In the context of a clinical trial, it provides a way whereby the trial data is combined with data from pilot studies, results of similar trials, subjective clinical opinion and other sources of information. Any decisions or conclusions made will then have been based on all available information.

In the context of the Phase II trials of gemcitabine in patients with metastatic NPC, the frequentist analysis estimates the true rates θ_*c*_ (for chemotherapy-naïve) and θ_*p*_ (for previously treated) as 28% and 48% respectively. The uncertainty associated with these estimates is quantified by the 95% CI. However, this CI does not, as is commonly understood, refer to an interval which contains θ (either θ_c_ or θ_p_) with a 95% probability. Instead, it refers to the interval that, should the trial be repeated again and again, will contain θ exactly 95% of the time. Since we are unlikely ever to repeat the trial again, this property is somewhat non-intuitive in nature. In addition, the frequentist estimate of the response rate and corresponding 95% CI, take no note of any external information.

In a Bayesian framework, θ is not treated as a fixed value, but is regarded as a variable with a probability distribution to be determined. This then allows the calculation of the probability that θ lies in a given interval. This enables simpler and more direct interpretations to be made regarding the results of the trial. For example, in Table 1, we give the posterior probability values of θ_c_ being less than *R*_0_, being between *R*_0_ and *R*_1_ and being greater than *R*_1_. In fact, we can calculate the probability values of θ_c_ being in any interval of interest, not just at those defined by *R*_0_ and *R*_1_. Thus, suppose we were interested in obtaining the final posterior probability, having started with the clinical prior, that θ_c_ exceeded 0.4. From Figure 2, this is the area under the Stage 2 posterior curve which exceeds θ_c_=0.4. This turns out to be 0.080, that is there is a probability of 8% that the true response proportion exceeds 0.4 or 40%.

The above are some of the possible advantages of using a Bayesian approach. However, there are also some difficulties. Foremost among these, is the ‘subjective’ nature of Bayesian approaches. Also criticised, is the perceived difficulty and relatively longer computational time required to carry out Bayesian computations as opposed to frequentist ones. Many articles have been written debating the pros and cons of Bayesian approaches and some of these are discussed by Berry and Stangl (1996) and Senn (2000).

Based on the response rates and their associated CIs, Foo *et al* (2002) concluded that gemcitabine has moderately high single-agent activity in NPC of the undifferentiated type whether as a first-line or as a salvage treatment. They comment that, while it appears that those who had prior treatment seemed to respond better to gemcitabine, this can possibly be explained by patient selection. Those who had prior chemotherapy had better performance status and lower disease bulk as evidenced by the fewer sites of distant metastases at accrual.

Formulated in Bayesian terms, the conclusions would be rather similar except perhaps that one would quote the respective probabilities that θ⩾*R*_1_ of λ_c_=0.372 and λ_p_=0.999 using the clinical priors. However, for the chemotherapy naïve group sceptical prior distribution the probability that θ_*C*_⩾0.3 is relatively small as λ_*C*_=0.156. Thus, there remains some concern with respect to efficacy and hence further study. In contrast, for the previously treated group, there is a large probability that θ_p_⩾0.2, here λ_p_=0.993. Gemcitabine is clearly recommended for further study in this situation.

In the particular circumstance of two similar trials running in parallel, we have illustrated how Bayesian methods could be used. In this case to allow the Stage 1 results from one trial to influence the decision to proceed to Stage 2 for the other. For didactic reasons, we have utilised the clinical prior only in this. Essentially, the (down-weighted) posterior distribution following the results of Stage 1 in one trial is combined with the posterior distribution calculated at the end of Stage 1 in the second trial. This combined posterior distribution is then used to calculate the probability that the true proportion of response is greater than a predefined threshold. In this example, we have set the down-weighting factor *k*=4 and the threshold probability λ=¼=0.25.

The latter was set at 0.25 deliberately to (just) allow (under this Bayesian monitoring) Stage C2 to be implemented. The calculated probability was 0.278 whereas without the down-weighted posterior following P1, it was 0.164 (Table 1, row 2). Had the enthusiastic prior been utilised the probability of 0.229 would still be less than the threshold λ to continue to Stage C2. Clearly had the threshold been set at λ=0.3, for example, the trial would not have continued.

The value of *k*=4 was chosen arbitrarily to reduce the standard error of the (frequentist) estimate of θ by 2. Fortuitously, this gave a value just above the threshold we had set – enabling the trial to continue in our theoretical setting. However, we have no direct experience to suggest a ‘better’ value. But, just as clinical, sceptical and enthusiastic priors may be obtained, then a range of values of *k* may be sought and utilised. For example, we could have asked the investigators, before the start of the trial, how relevant (on a scale of 0 to 1) they felt information from the previously treated group would be to the chemotherapy-naïve group, or vice-versa. We could then take the average of all their opinions, and use the inverse of this as our value of *k*. Alternatively, we could search the literature for other studies (if necessary, involving other tumour types or other treatment regimes) that give response rates for chemotherapy-naïve and previously treated patients. These need not come from the same study. We can then evaluate the ratio of the two rates, and use the average of these as *k*.

It may be argued that our setting is rather unique, two parallel trials using the same regimen and in the same disease. However, we would claim this is often the case. For example, in the recent literature there are three related Phase II trials of single agent gemcitabine in breast cancer – related in the sense of both overlapping authorship and type of disease (metastatic and/or advanced) (Carmichael *et al*, 1995; Possinger *et al*, 1999; Schmid *et al*, 1999). However, the methodology is equally applicable if the (down-weighted) prior had been obtained from information external to the investigating team – perhaps from the newly available literature.

Thus far, we have demonstrated how Bayesian methodology could be usefully applied to the interpretation of Phase 2 trials already designed using the two-stage Simon minimax design. However, suppose instead we wished to prospectively use a Bayesian approach to design a Phase 2 clinical trial.

The two-stage Simon minimax design requires specification of four items, *R*_0_ and *R*_1_ as well as the Type I and II error rates. The two stages (*n*_1_ + *n*_2_=*N*) are then chosen with the smallest *N* possible to satisfy the specified error rates. Designs for a range of values have been tabulated by Simon (1989) and others. In its simplest form, where no external information is going to be available at the end of Stage 1, the Bayesian design requires specification of a prior distribution for θ and a value for λ.

Several authors have discussed the issue of specifying a prior distribution for a Phase II trial. For example, Heitjan (1997) propose standard forms for sceptical and enthusiastic priors, while Thall and Simon (1994), Tan and Machin (2002) and others propose forms for clinical and reference priors. All the proposed methods involve eliciting appropriate summary values from the investigators and defining a prior distribution based on these. This is similar to what we have done for our clinical prior, with the clinician-elicited values of *R*_0_ and *R*_1_ being used to construct the prior distribution. As far as eliciting a full prior distribution is concerned, most of the work in this area has been in the context of Phase III trials (see for example Parmar *et al*, 1994). However, similar approaches could be used for Phase II trials.

As for specifying λ, Tan and Machin (2002) recommend that this should be chosen to give sample sizes which are practical for Phase II trials. Choosing a larger value of λ will increase the ‘power’ of the design, but at the cost of a larger sample size. There is a need to trade-off wanting a high λ value with keeping the sample size reasonably small. This trade-off also happens in frequentist designs, where the sample size is traded off against the Type I and II errors.

 More generally, values of *R*_0_ and *R*_1_ (from which a prior distribution may be derived) as well as two threshold values, λ_0_ and λ_1_, corresponding to the ‘interim’ and ‘final’ thresholds would need to be specified. The overall sample size would then be selected so as to allow for an expected posterior probability of at least λ_1_ that θ exceeds *R*_1_. The Stage 1 sample size would be selected so as to allow for an expected posterior probability of at least λ_0_, where λ_0_<λ_1_. Note that if we fix λ_0_=*c*λ_1_ for some predefined constant *c*<1, then only one threshold value needs to be specified.

However, these designs too would have to be tabulated or at least specialised software made available for general use. Work on tabulating the designs has begun (see Tan and Machin, 2002) and we plan to implement these alongside the Simon minimax design and other proposed Bayesian Phase 2 designs before making a judgement on their practical usefulness. The additional possibility of using external information at the decision point to continue to Stage 2 can then be explored also.

In general, we believe that Bayesian methods have much to offer the design, analysis and hence reporting of Phase II clinical trials. These parallel their use in Phase III trials as has been advocated by Spiegelhalter *et al* (1994) and others. In particular we have adopted their concepts of sceptical and enthusiastic priors for our approach.
